# Near-Unity and Narrowband Light Absorption in Mid-Infrared α-MoO_3_-Based Metasurfaces

**DOI:** 10.3390/nano16211388

**Published:** 2026-10-29

**Authors:** Didi Song, Wei Li, Lijia Wan, Yufang Liu, Xiaohu Wu, Kun Yu

**Affiliations:** 1 School of Science, Jiangxi University of Water Resources and Electric Power, Nanchang 330099, China; didisong1025@163.com (D.S.);; 2 Henan Key Laboratory of Infrared Materials & Spectrum Measures and Applications, School of Physics, Henan Normal University, Xinxiang 453007, China; 3 School of Integrated Circuits, Hubei University, Wuhan 430062, China; 4 Yangtze Laboratory, Wuhan 430205, China; 5 Thermal Science Research Center, Shandong Institute of Advanced Technology, Jinan 250100, China

**Keywords:** Mid-infrared, narrowband light absorption, α-MoO_3_, metasurfaces

## Abstract

Accessing mid-infrared narrowband absorption is crucial for applications such as spectroscopic analysis, infrared imaging and optical sensing. Here, we investigate the interaction between light and α-phase molybdenum trioxide (α-MoO_3_) nanocavities, uncovering pronounced and highly tunable resonant responses across the hyperbolic transition regime. Moving beyond standard phonon polariton excitations, our results reveal that the elevated refractive index of α-MoO_3_ beyond the Reststrahlen band promotes strong electromagnetic confinement and enhanced light-matter coupling in subwavelength nanostructures across the 12–13 μm spectral window. Analysis of the electric-field distributions reveals a hybrid collective mode arising from the interaction between Fabry–Perot cavity modes and geometry-dependent Mie-type resonances, leading to absorption approaching unity together with spectrally sharp resonances exhibiting quality factors exceeding 100 near the transverse-optical phonon frequency of α-MoO_3_. These findings provide novel insights for the development of compact mid-infrared photonic structures for tailoring thermal emission and enhancing electromagnetic coupling in α-MoO_3_-based nanophotonic platforms.

## 1. Introduction

Efficient manipulation and absorption of light are essential for advancing micro- and nano-optoelectronic technologies. Mid-infrared (MIR) light absorption has found extensive applications in photodetection [1,2], gas sensing [3,4], infrared thermal imaging [5,6], and medical diagnostics [7,8]. These applications often require narrowband spectral responses, highlighting the importance of developing highly efficient narrowband absorbers. Resonant approaches to light manipulation and absorption include gratings [9], photonic crystals, and dielectric resonators [10,11], with material platforms ranging from metals such as gold [12] and dielectrics such as zinc sulfide [13] and germanium [14] to polar dielectrics. Owing to their intrinsic phonon resonances, polar dielectrics—such as silicon carbide (SiC) and hexagonal boron nitride (hBN)—offer distinctive opportunities for manipulating electromagnetic fields and optical coupling in the MIR regime, attracting considerable attention in recent years [13,14,15,16,17,18,19,20,21]. For instance, overlapping electric and magnetic Mie resonances have enabled narrowband absorption in SiC near 13 μm [15], while Fabry–Perot (FP)-like and Mie-like responses have been investigated in hBN in the 7–8 μm region [16]. More generally, coupling between optical phonons and photonic modes can give rise to hybrid light-matter states, expanding the possibilities for infrared optoelectronic applications.

Recently, polar van der Waals crystals that support phonon polaritons have attracted widespread attention for their remarkable capability to confine infrared light at subwavelength scales, thereby enabling precise light guidance and manipulation at the nanoscale [22,23]. In particular, α-phase molybdenum trioxide (α-MoO_3_) has emerged as a natural polar van der Waals crystal, characterized by three orthogonal crystal axes, three Reststrahlen bands (RBs), and the ability to support highly confined hyperbolic phonon polaritons in the MIR range [24,25,26]. Studies have shown that phonon polaritons supported by α-MoO_3_ films exhibit remarkable electromagnetic responses in the infrared regime, featuring extreme field localization [27], low-loss polaritonic propagation, and strongly anisotropic optical coupling [26,28]. These characteristics make α-MoO_3_ a promising platform for a variety of applications, including super-resolution imaging [26], directional thermal energy transfer [29], molecular vibrational sensing, and photodetection [30,31]. Previous research on α-MoO_3_ has predominantly concentrated on its in-plane hyperbolic characteristics. The transition across the RB (i.e., from hyperbolic to highly anisotropic dielectric) and the role of strong material dispersion on the dynamics of excitations in micro- and nanostructured α-MoO_3_ remain unexplored. In particular, near the transverse optical (TO) phonon resonance associated with the [100] direction, large positive values of Re(*ε_x_*) provide a high refractive index for light polarized along this direction, supporting resonances in structures with subwavelength thicknesses [15]. This phonon-induced dielectric dispersion makes α-MoO_3_ a suitable platform for investigating geometry-tunable narrowband absorption in the 12–13 μm region.

In this work, we examine light-matter interactions within subwavelength α-MoO_3_ metasurfaces operating near the TO phonon frequency associated with the upper RB as α-MoO_3_ evolves from a hyperbolic to a dielectric regime. Under transverse magnetic (TM) polarization, the proposed α-MoO_3_ nanostructure is shown to support tunable near-perfect absorption in the MIR range, particularly where the dielectric permittivity along the [100] crystallographic direction remains positive (i.e., *ε_x_* > 0). The evolution of the resonant response across the upper Reststrahlen band and the hyperbolic-to-dielectric transition is systematically investigated as a function of nanocavity geometry. Particular attention is devoted to the interplay among hyperbolic phonon polaritons, FP-like resonances, Mie-type resonances, and geometric confinement effects. Finally, we demonstrate that this subwavelength structure supports multiple high-quality resonances, with Q factors exceeding 100, that can be precisely tuned through the α-MoO_3_ nanostructure.

## 2. Theoretical Model and Method

**Figure 1:**
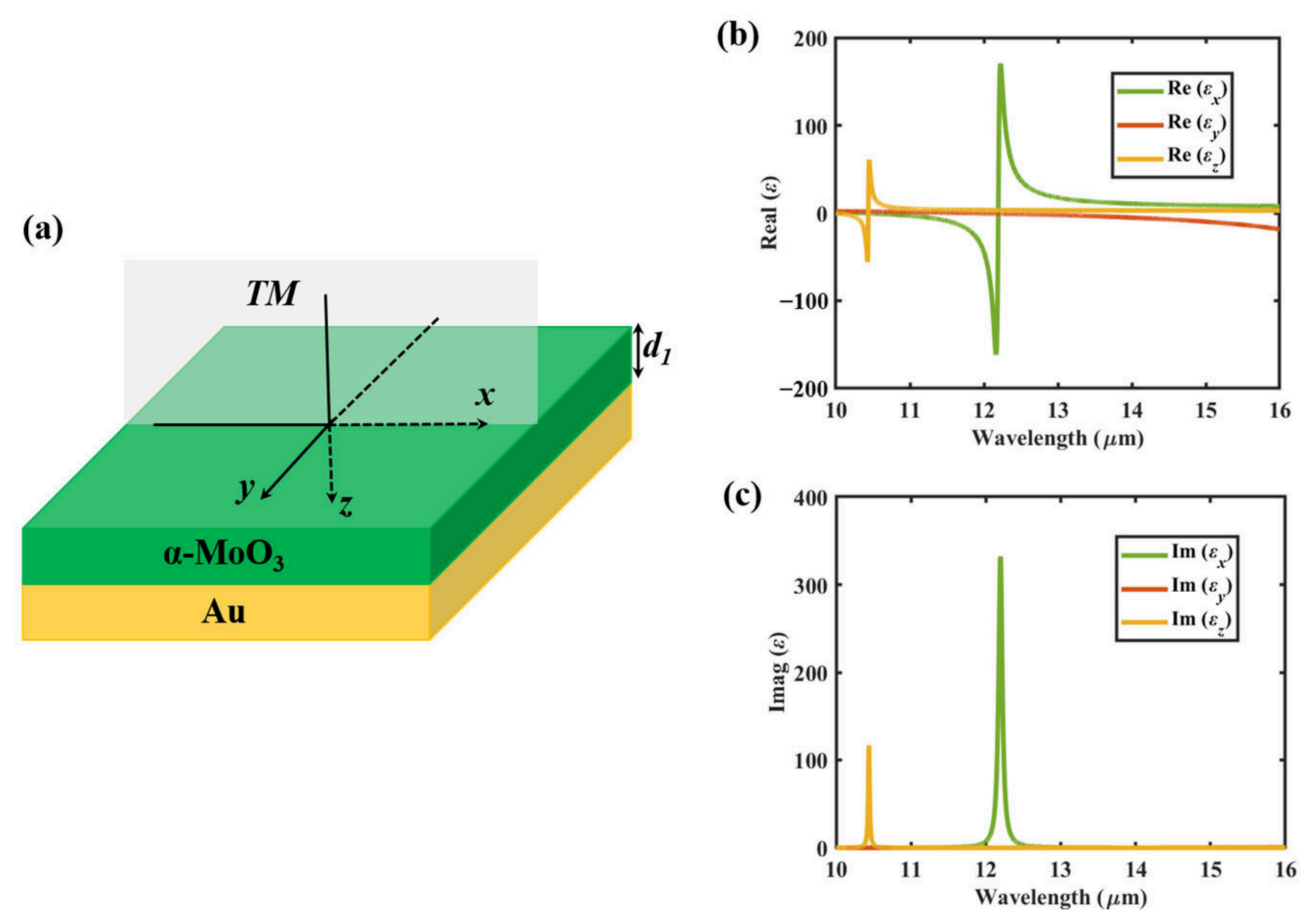
(**a**) Schematic illustration of the proposed planar α-MoO_3_ nanocavity (the arrows indicate the coordinate axes (*x*, *y*, *z*), the polarization direction of the incident light (TM), and the thickness of the α-MoO_3_ layer (*d*_1_)). (**b**,**c**) Real and imaginary components of the dielectric permittivity of α-MoO_3_ along its principal crystallographic axes over the 10–16 μm spectral range.

Figure 1a illustrates the proposed planar FP nanocavity composed of an α-MoO_3_ thin film and a gold (Au) substrate. The permittivity of the Au substrate is derived from the Drude model [32], with a fixed thickness of 0.2 μm, which exhibits near-perfect reflection in the MIR range, thereby enhancing the interaction between light and the α-MoO_3_ film. The FP cavity is formed within the α-MoO_3_ film between the air/α-MoO_3_ and α-MoO_3_/Au interfaces. The Au layer acts as a back reflector, while optical propagation and the associated phase accumulation occur within the α-MoO_3_ film. Reflections at the two interfaces give rise to thin-film interference, allowing FP resonances at subwavelength film thicknesses owing to the high refractive index near the relevant TO phonon resonance [15,33]. The total absorptance of the Au-backed structure is calculated as *A* = 1 − *R*, where *R* is the reflectance and transmission through the optically thick Au film is neglected. This absorptance includes losses in both α-MoO_3_ and Au, whose individual contributions have not been separately quantified. Unless otherwise specified, the reported spectra are calculated at normal incidence with the incident electric field aligned along the [100] crystallographic direction (the *x*-axis). The thickness of α-MoO_3_ is denoted as *d*_1_. Given that optical phonons primarily influence the optical properties of α-MoO_3_ in the MIR range, its complex permittivity can be effectively modeled using the Lorentz framework:

(1) $$\varepsilon_{j}=\varepsilon_{\infty ,j}\left(1+\frac{\omega_{LO,j}^{2}-\omega_{TO,j}^{2}}{\omega_{TO,j}^{2}-\omega^{2}+i\Gamma_{j}\omega }\right),j=x,y,z.$$

where $\varepsilon_{\infty ,j}$ and $\Gamma_{j}$ denote the high-frequency dielectric constant and phonon damping coefficient, respectively, while $\omega_{LO,j}$ and $\omega_{TO,j}$ denote the longitudinal optical (LO) and TO phonon frequencies that define the boundaries of the RB along the corresponding crystallographic direction *j*. The specific parameters for these variables are detailed in reference [34]. Here, *x*, *y* and *z* represent the three principal axes of the crystal, aligning with the crystallographic directions [100], [001] and [010] of α-MoO_3_, respectively. Figure 1b,c display the real and imaginary parts of the permittivity of α-MoO_3_ along its three principal crystal axes within the wavelength range of 10–16 μm. One can see that Re(εx) is negative in the 10.3–12.19 μm range. As the TO phonon frequency is approached, α-MoO_3_ exhibits a pronounced increase in refractive index, leading to the excitation of subwavelength dielectric resonances and Fabry–Perot cavity behaviour within the film.

In this study, we employed the transfer matrix method to numerically compute the absorption spectrum of the planar α-MoO_3_ nanocavity. Additionally, we utilized the Wave Optics module in COMSOL Multiphysics 6.1 to perform finite element simulations and analyze the optical properties of the α-MoO_3_ disk array. By integrating the transfer matrix method and finite element methods, we achieved a more comprehensive understanding of the optical performance of α-MoO_3_, providing a theoretical foundation and technical support for its applications in optical devices and sensors.

## 3. Results

**Figure 2:**
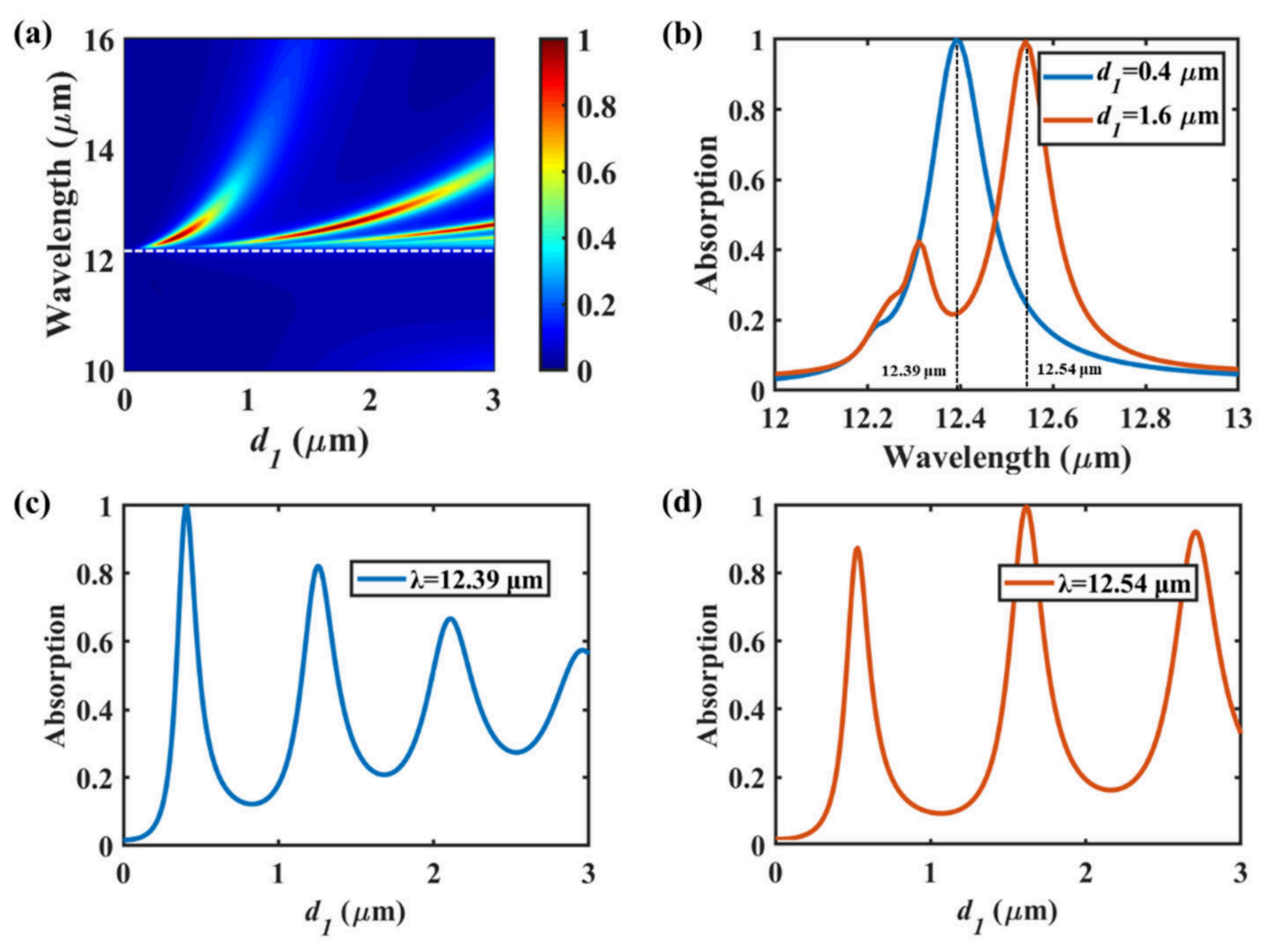
Nanocavity of α-MoO_3_ thin films. (**a**) Two-dimensional absorption map of the nanocavity for varying α-MoO_3_ thickness. The white dashed line in (**a**) denotes the onset of RB near the TO phonon energy, where the sign of *ε_x_* changes. (**b**) Absorption spectra for selected α-MoO_3_ thicknesses (*d*_1_) of 0.4 μm and 1.6 μm. Absorption variation with α-MoO_3_ thickness at wavelengths of (**c**) 12.39 μm and (**d**) 12.54 μm.

We initiate our analysis by investigating the resonant interaction between photons and phonons within planar α-MoO_3_ nanocavities. Figure 2a depicts the evolution of the nanocavity absorption spectrum with increasing α-MoO_3_ thickness under TM polarization. Multiple resonant absorption branches are observed near a wavelength of 12.19 μm (indicated by the white dashed line). As *d*_1_ increases, the resonance wavelength undergoes a redshift, consistent with the resonance evolution expected for a FP cavity operating in a regime of normal dispersion. Figure 2b shows the absorption spectra of α-MoO_3_ nanocavities with thicknesses of 0.4 μm and 1.6 μm, extracted from Figure 2a. When *d*_1_ is 0.4 μm, the nanocavity exhibits an almost complete absorption response (A ≈ 0.99) occurring at 12.39 μm, accompanied by a high-quality factor (Q ≈ 83), crucial for designing narrowband infrared emitters and filters. As the thickness of α-MoO_3_, *d*_1_, increases to 1.6 μm, the absorption spectrum reveals two prominent absorption peaks at wavelengths of 12.30 μm and 12.54 μm, corresponding to high-order and low-order vibrational modes within the FP cavity. Notably, at a wavelength of 12.54 μm, the absorption reaches 0.99 with the Q approximately 105. This near-unity absorptance indicates strong suppression of reflection from the Au-backed structure at resonance. Additionally, the observed nearly perfect light absorption is accompanied by narrowband characteristics, attributed to the strong dispersion of the dielectric constant near the TO resonance of α-MoO_3_. Taking wavelengths of 12.39 μm and 12.54 μm as examples, the absorption as a function of the α-MoO_3_ thickness is shown in Figure 2c,d. The results indicate multiple peaks in the absorption spectra of α-MoO_3_, attributed to FP resonances. The resonance period is approximately 0.85 μm at a wavelength of 12.39 μm and 1.09 μm at a wavelength of 12.54 μm. At a wavelength of 12.39 μm, the first-order FP resonance is supported by an α-MoO_3_ thickness of 0.41 μm, whereas a thickness of approximately 1.62 μm is required to sustain the second-order resonance at 12.54 μm. The predicted resonance conditions are in close agreement with the numerical calculations. Therefore, the resonant energy of photon-phonon interactions in planar α-MoO_3_ nanocavities is primarily influenced by the thickness of the α-MoO_3_ film.

**Figure 3:**
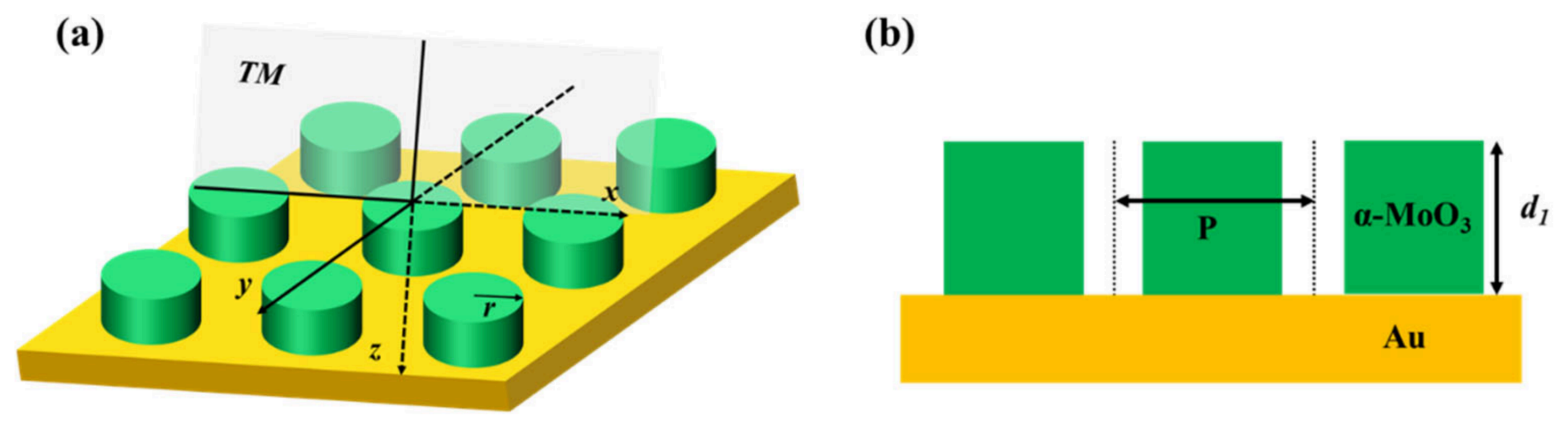
(**a**) Schematic illustration of the α-MoO_3_ disk array microstructure (where *r* denotes the disk radius). (**b**) Side view of the microstructure (where *P* denotes the array period and *d*_1_ denotes the thickness of the α-MoO_3_ layer).

Next, we extend planar α-MoO_3_ films to patterned arrays of α-MoO_3_ disks, aiming to explore how nanoscale structures can be designed to manipulate the dispersion properties of the material. Figure 3a depicts a schematic of the patterned α-MoO_3_ microstructure, consisting of a periodic arrangement of α-MoO_3_ disk arrays and continuous Au films. The thickness of the α-MoO_3_ disks is denoted as *d*_1_, with a fixed thickness of 0.2 μm for the Au layer to ensure structural opacity, as shown in Figure 3b. The radius of the α-MoO_3_ disks is denoted as *r*, following a periodic distribution in both the *x* and *y* directions, with a period (*P*) of 8.4 μm. Our goal is to investigate the resonant modes of α-MoO_3_ in the dielectric constant (*ε_x_* > 0) under TM-polarized light, and to elucidate the evolution of light-matter interactions through the hyperbolic-to-dielectric transition near the TO phonon frequency.

**Figure 4:**
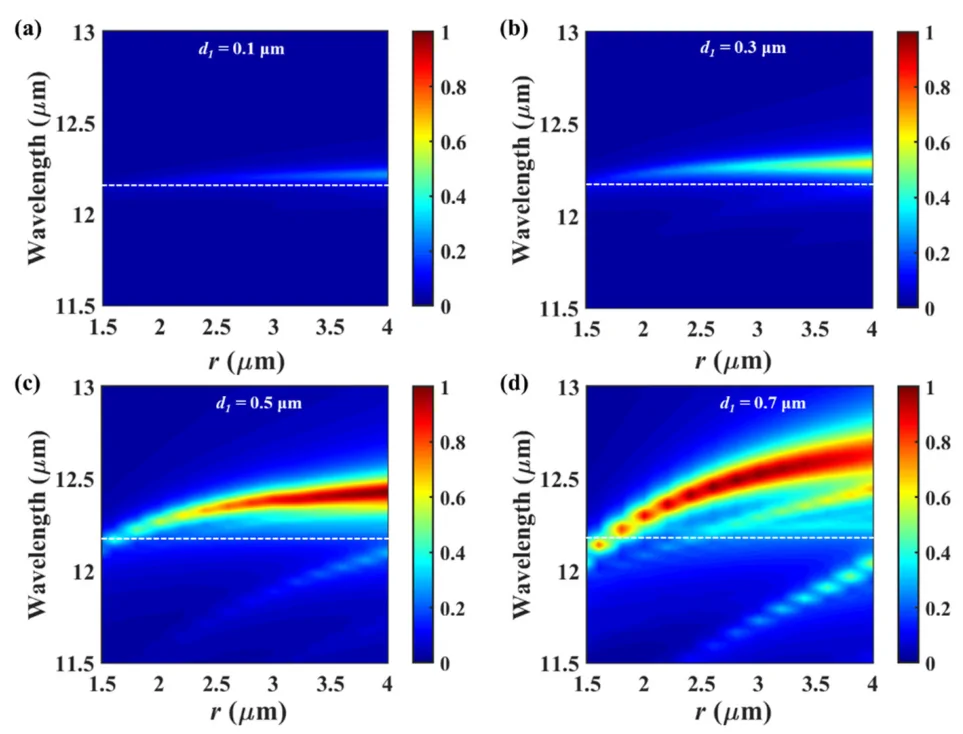
Broadband tunable strong MIR light–material interactions. Calculated absorption spectra in α-MoO_3_ disk arrays with varying disk radius for (**a**) *d*_1_ = 0.1 μm, (**b**) *d*_1_ = 0.3 μm, (**c**) *d*_1_ = 0.5 μm, and (**d**) *d*_1_ = 0.7 μm films, respectively. Dashed lines in each plot denote the onset of the RB near the TO phonon energy, marking the transition where *ε_x_* changes sign.

Firstly, we performed detailed numerical simulations to calculate the variation in light absorption along the RB edge hyperbolic transition with changes in α-MoO_3_ film thickness. Figure 4 presents a color map of the absorption spectra of α-MoO_3_ disk arrays as a function of disk radius for various α-MoO_3_ thicknesses. For thinner α-MoO_3_ films (*d*_1_ = 0.1 μm and *d*_1_ = 0.3 μm, see Figure 4a,b), only one resonance mode is observed within the range of *ε_x_* > 0. As the thickness of α-MoO_3_ increased, this resonance mode shifted to longer wavelengths, consistent with the results observed for the FP nanocavity in Figure 2. For thicker α-MoO_3_ films, additional higher-order resonance modes can be observed, as shown in Figure 4d. These modes enable strong and tunable light absorption in subwavelength structured films over the wavelength range of 11.5 μm to 13 μm. These findings provide critical insights for the engineering control of absorption and emissivity in subwavelength structured films, offering new directions for the design and optimization of optical devices. To further investigate the interaction between light and α-MoO_3_, we considered two different α-MoO_3_ film thicknesses: an ultrathin film with *d*_1_ = 0.3 μm and a thicker film with *d*_1_ = 0.7 μm. The thicker film is expected to exhibit a broader range of excitation phenomena.

**Figure 5:**
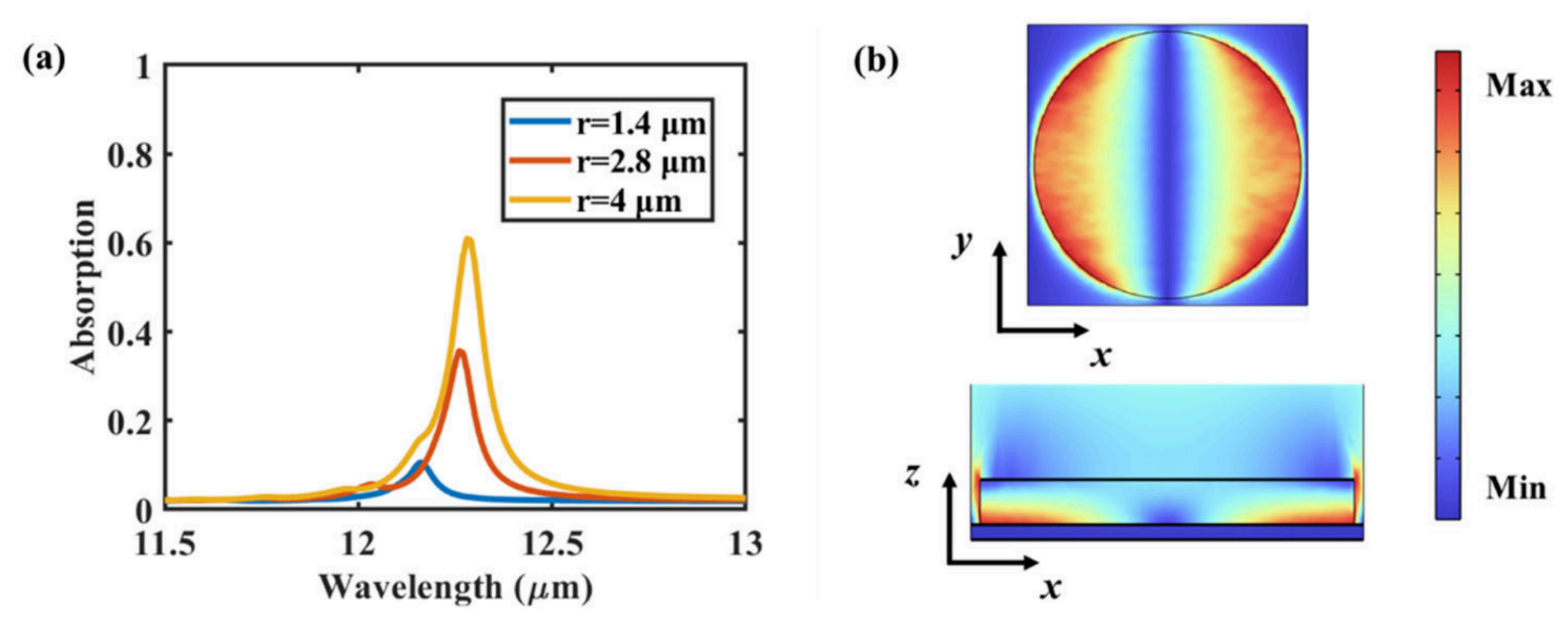
Study of thin α-MoO_3_ disk arrays with *d*_1_ = 0.3 μm. (**a**) Absorption spectra of α-MoO_3_ disk arrays with different radii. (**b**) With a radius of 4 μm, the excitation field intensity of the α-MoO_3_ disk array at the resonance wavelength of 12.28 μm is analyzed in the *x*–*y* plane and *x*–*z* plane.

We investigated the absorption spectra of patterned α-MoO_3_ nanocavities with *d*_1_ = 0.3 μm for various disk radius under TM polarization. In Figure 5a, a single resonant mode is observed as the radius of the α-MoO_3_ disks increases from 1.4 μm to 4 μm within the wavelength range of 12.19–13 μm (*ε_x_* > 0), with a slight redshift in the resonance wavelength. When *r* is 4 μm, the nanocavity exhibits an absorption of 0.6 at a wavelength of 12.28 μm, achieving a Q factor of 123. This excitation exhibits FP-like characteristics, qualitatively resembling the response observed in the planar α-MoO_3_ nanocavity (Figure 2). To examine the field distribution of this resonance, Figure 5b shows the electric-field distributions in the *x*–*y* and *x*–*z* planes at 12.28 μm. In the *x*–*y* plane, edge effects significantly enhance the electric field at the disk edges. In the *x*–*z* plane, the electric field is mainly confined within the α-MoO_3_ cavity, effectively trapping light and enhancing light-matter interactions. The edge-field enhancement also indicates the influence of the finite lateral geometry on this FP-like response. Although this resonance provides a narrow spectral response with a Q factor of 123, its peak absorptance of 0.6 indicates that this particular configuration does not achieve near-unity absorption. By adjusting the radius of the α-MoO_3_ disks, the optical resonance characteristics can be precisely controlled. Under TM polarization, the unique tuning characteristics of the absorption spectra and electric field distribution provide new insights for the design and application of nanophotonics and infrared optical devices.

**Figure 6:**
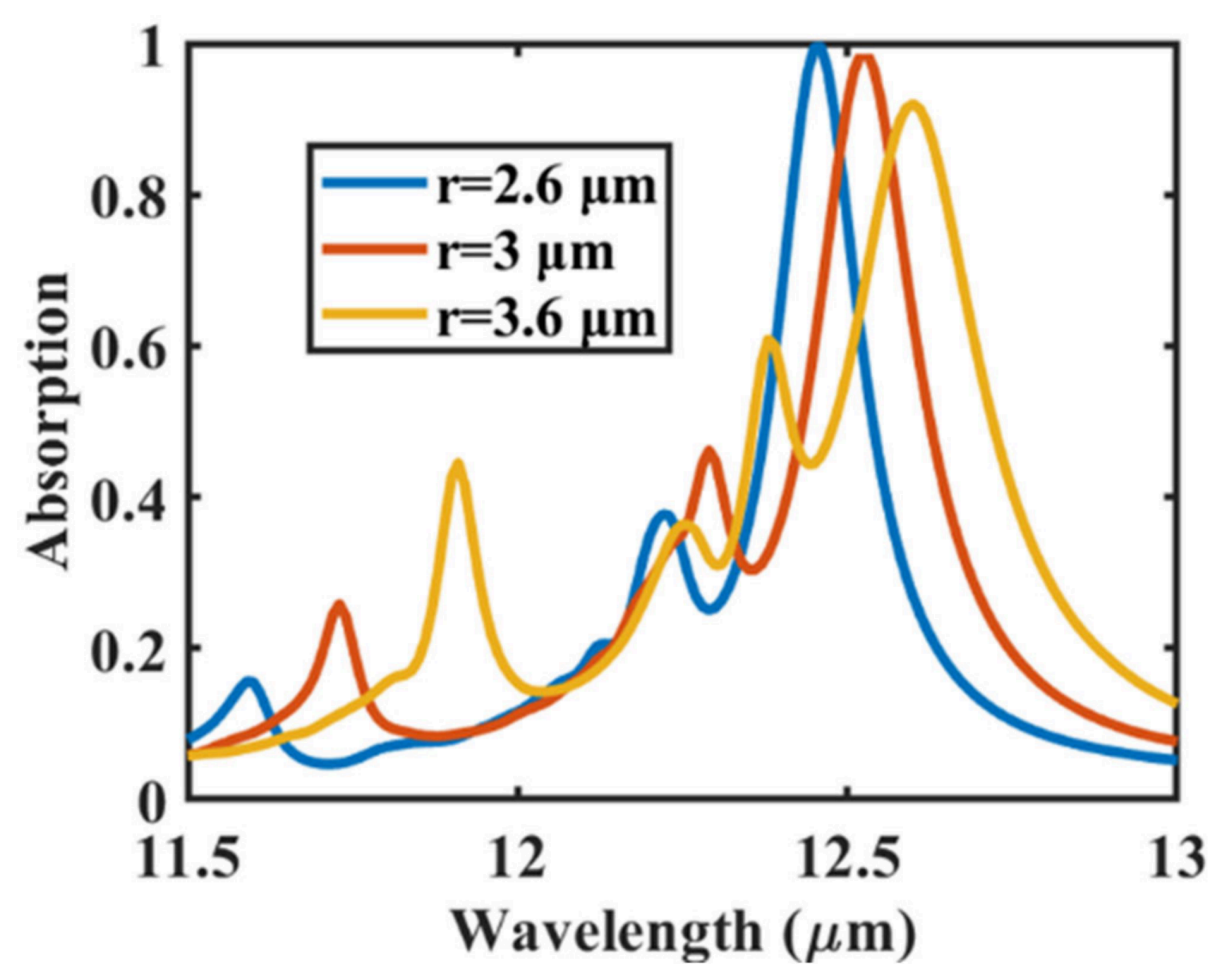
Absorption spectra of α-MoO_3_ disk arrays with radii of 2.6 μm, 3 μm, and 3.6 μm for a fixed *d*_1_ of 0.7 μm.

Next, we investigated the absorption spectra of patterned α-MoO_3_ nanocavities with *d*_1_ = 0.7 μm as the radius varied, as shown in Figure 6. Unlike the case with *d*_1_ = 0.3 μm, the thicker α-MoO_3_ film exhibits multiple resonant absorption features associated with phonon-polaritonic excitations within the RB region (11.75–12.19 μm). Simultaneously, a set of resonance absorption peaks strongly dependent on the disk radius is observed when *ε_x_* > 0 (within the wavelength range of 12.19 to 13 μm). The phonon polariton branches can also be clearly observed in Figure 4d, exhibiting strong dispersion with varying disk radius. This behavior can be explained by the dispersion of phonon polaritons in the planar α-MoO_3_ film and the hyperbolicity of the optical dispersion of α-MoO_3_ within the RB. Specifically, the disk radius *r* determines the effective wavevector of the excited phonon polariton, *k_pp_*, which relates to *k_pp_* ∝ 1/*r* and possesses in-plane directionality, i.e., *k_x_*∼*k_pp_* [23]. Therefore, a smaller disk radius implies larger planar wavevectors *k_x_* for the excited modes. According to the dispersion relation, these modes deviate from the resonance wavelength of 12.19 μm, extending into the RB region, when |*ε_x_*| approaches 0. Conversely, a larger disk radius necessitates a smaller *k_pp_*, achievable under larger |*ε_x_*|. Moreover, a larger disk radius can also excite higher-order phonon polariton modes, leading to a redshift in resonance wavelength as the disk radius increases.

**Figure 7:**
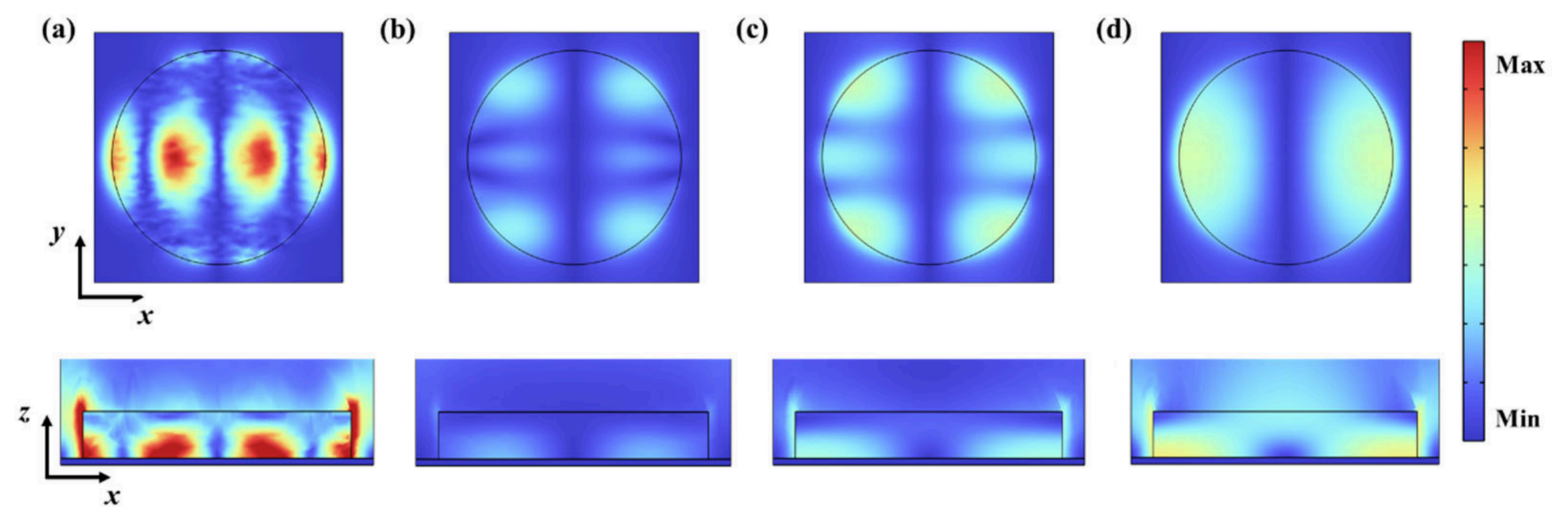
Electric field distributions of α-MoO_3_ disk arrays (*d*_1_ = 0.7 μm, *r* = 3.6 μm) at resonance wavelengths of (**a**) 11.9 μm, (**b**) 12.25 μm, (**c**) 12.38 μm, and (**d**) 12.6 μm.

Figure 7 illustrates the excitation field distribution of α-MoO_3_ disk arrays with *d*_1_ = 0.7 μm and *r* = 3.6 μm at resonant wavelengths of 11.9 μm, 12.25 μm, 12.38 μm, and 12.6 μm. In Figure 7a, a clear observation of high-*k* mode excitation inside the disk is evident. Such modes typically occur within the hyperbolic band (where the dielectric constant along the *x*-axis of α-MoO_3_ is negative), indicating highly localized electric field enhancement within this region due to the disk structure. Figure 7d displays the field strength distribution at the resonant wavelength of 12.6 μm. These modes occur in the region where the dielectric constant along the *x*-axis of α-MoO_3_ is positive, resembling FP resonances and exhibiting characteristics akin to standing wave modes in thin films. The field patterns at resonant wavelengths of 12.25 μm and 12.38 μm (Figure 7b and Figure 7c, respectively) distinctly differ from those at 12.6 μm (Figure 7d), displaying characteristics similar to Mie-like resonances. These distinct field patterns suggest additional effects of lateral confinement introduced by the finite disk geometry. The FP-like and Mie-like designations describe qualitative resonance characteristics in this anisotropic, metal-backed structure, where thin-film interference and lateral confinement can both influence the response. For the near-unity narrowband absorption functionality considered here, resonance selection should consider both peak absorptance and spectral linewidth. Neither the FP-like nor the Mie-like designation alone determines suitability for this objective. The spectral evolution and field distributions illustrate the combined influence of the α-MoO_3_ dielectric response and structural geometry on the optical response, providing guidance for the design of α-MoO_3_-based resonant absorbers.

## 4. Conclusions

In summary, the metasurface design provides a platform for investigating the strong coupling of infrared light with phonons in α-MoO_3_. Extending beyond conventional studies of phonon polariton branches, our work demonstrates that the high refractive index of α-MoO_3_, coupled with its extreme dispersion in the *ε_x_* > 0 region, enables the effective utilization of subwavelength structures. Narrowband resonances with Q factors up to 123 in the 12.2–13.5 μm range exhibit nearly uniform absorption, offering potential applications in infrared sources, thermal management, and spectroscopy. Furthermore, exploiting the dispersion and anisotropy of α-MoO_3_ offers new degrees of freedom for designing infrared radiation across multiple spectral bands.

## Data Availability

The participants of this study did not give written consent for their data to be shared publicly, so due to the sensitive nature of the research, supporting data is not available.

## Abbreviations

The following abbreviations are used in this manuscript:

α-MoO_3_	α-phase molybdenum trioxide
MIR	Mid-infrared
hBN	hexagonal boron nitride
RB	Reststrahlen band
TO	Transverse Optical
TM	Transverse Magnetic
FP	Fabry–Perot
LO	Longitudinal Optical
